#  A family with Wagner syndrome with uveitis and a new versican mutation

**Published:** 2013-09-26

**Authors:** Pierre-Raphaël Rothschild, Antoine P. Brézin, Brigitte Nedelec, Cyril Burin des Roziers, Tiffany Ghiotti, Lucie Orhant, Mathieu Boimard, Sophie Valleix

**Affiliations:** 1 AP-HP, Groupe Hospitalier Cochin-Hôtel-Dieu, Service d’ophtalmologie, Université Paris Descartes, Sorbonne Paris Cité, Paris, France; 2 INSERM, Centre de Recherche des Cordeliers, Paris, France; 3 AP-HP, Groupe Hospitalier Cochin-Hôtel-Dieu, Laboratoire de Biochimie et Génétique Moléculaire, Université Paris Descartes, Sorbonne Paris Cité, Faculté de Médecine Paris, France

## Abstract

**Purpose:**

To report the clinical and molecular findings of a kindred with Wagner syndrome (WS) revealed by intraocular inflammatory features.

**Methods:**

Eight available family members underwent complete ophthalmologic examination, including laser flare cell meter measurements. Collagen, type II, alpha 1, versican (*VCAN*), frizzled family receptor 4, low density lipoprotein receptor-related protein 5, tetraspanin 12, and Norrie disease (pseudoglioma) genes were screened with direct sequencing.

**Results:**

The index case was initially referred for unexplained severe and chronic postoperative bilateral uveitis following a standard cataract surgery procedure. Clinical examination of the proband revealed an optically empty vitreous with avascular vitreous strands and veils, features highly suggestive of WS. The systematic familial ophthalmologic examination identified three additional unsuspected affected family members who also presented with the WS phenotype, including uveitis for one of them. We identified a novel c.4004–6T>A nucleotide substitution at the acceptor splice site of intron 7 of the *VCAN* gene that segregated with the disease phenotype.

**Conclusions:**

We present a family with WS with typical WS features and intraocular inflammatory manifestations associated with a novel splice site *VCAN* mutation. Beyond the structural role in the retinal-vitreous architecture, versican is also emerging as a pivotal mediator of the inflammatory response, supporting uveitis predisposition as a clinical manifestation of WS.

## Introduction

Wagner syndrome (WS; OMIM_143200) is a rare autosomal dominant vitreoretinopathy characterized by an optically empty vitreous along with avascular veils and strands at the equatorial vitreoretinal interface. However, other clinical manifestations have been described in patients with WS, including retinal detachment, myopia, presenile cataract, night blindness with progressive chorioretinal atrophy, extensive retinal pigment clumping, ectopic foveae, inverted papilla, uveitis, and glaucoma [1-9]. Thus, there is no doubt that the full spectrum of the WS phenotype must be further refined. The clinical diagnosis of this ocular disease remains a challenge because the clinical presentation, expressivity, and severity vary among patients, resulting in the disease often being overlooked or misdiagnosed.

To date, no more than 13 families with WS (including the family reported here) have been characterized at the molecular level worldwide. All WS disease mutations described thus far are heterozygous nucleotide substitutions occurring at the splice acceptor of intron 7 or splice donor site of intron 8 of the versican (*VCAN*) gene, also known as chondroitin sulfate proteoglycan-2 (CSPG2), leading to aberrant splice products and/or to an imbalanced quantitative ratio of the four *VCAN* transcript variants [2-13]. To improve clinical diagnosis and to gain insights into the understanding of this complex disease, more clinical and molecular data are needed from families with WS. Herein, we describe a family with WS with a novel splice mutation in the *VCAN* gene, revealed by chronic intraocular inflammatory features.

## Methods

### Clinical characterization

Informed written consent from eight available family members without extraocular disease (4 females and 3 males; age range, 13-72 years) was obtained before they donated blood samples at Cochin hospital. The study followed the tenets of the Declaration of Helsinki and was approved by the ethics review committee of the Institutional Review Board of Cochin Hospital (Paris, France). Complete ophthalmologic examinations were performed by the same ophthalmologist (P.R.R.) on eight selected affected and unaffected family members (individuals I:2; II:1, II:2, II:3, II:4, II:5; III:1, III:2). The anterior chamber flare of all individuals was quantified with the Kowa FM 500 flare meter (Kowa, Tokyo, Japan), and the dilated fundus was photographed using the Canon fundus camera with Megaplus model 1.4 digital imaging system (Canon, Tokyo, Japan). Kinetic perimetry, tested with the Goldmann perimeter (Haag-Streit AG, Bern, Switzerland), and full-field electroretinography, with the MonElec2 Monitor Model (Metrovision, Pérenchies, France), were performed on the proband.

### Molecular analysis

DNA was extracted from venous blood samples collected by peripheral venepuncture in 2×15 ml EDTA tubes. The blood samples were stored at 2–8 °C before processing, using the QIamp DNA Mini Kit (Qiagen, Valencia, CA). Primers for PCR and sequencing were designed to target all coding sequences, intron-exon boundaries, and 5′- and 3′- untranslated regions of the *VCAN*, frizzled family receptor 4 (*FZD4*), Norrie disease (pseudoglioma) (*NDP*) genes as we previously reported [4]. Primers for tetraspanin 12 (*TSPAN12*) and low density lipoprotein receptor-related protein 5 (*LRP5*), exons were designed as reported by others [14,15]. Primers for Collagen, type II, alpha 1 (*COL2A1*), genes were designed using the Primer3 program (Appendix 1). Sequencing reactions were performed in an automatic genetic analyzer (ABI PRISM 3100 genetic analyzer; Applied Biosystems, Foster City, CA), using the BigDye Terminator Cycle Sequencing Kit (DNA sequencing kit; Applied Biosystems).

To determine the pathogenicity of the identified nucleotide changes, we combined data from familial segregation analysis, a control population of 200 unrelated individuals, the Single Nucleotide Polymorphism database (dbSNP), and bioinformatics tools such as Polyphen and Sorting Intolerant From Tolerant (SIFT), which are usually efficient to determine the predicted effects of coding non-synonymous variants.

### In silico versican transcript analyses

The following splice site prediction software were used: Human Splicing Finder [16], the MaxEnt Scan algorithm [17,18], NNSplice [19], NetGen2 [20,21], and ESEfinder [22,23]. The effect of the novel variant (c.4004–6T>A) on the strength of the canonical acceptor splicing site of intron 7 was compared to that of the reference sequence of the *VCAN* gene (Reference NG_012682) as well as to all WS disease-causing splicing mutations published to date.

## Results

### Ocular phenotypes

#### Individual II:1

**Figure 1:**
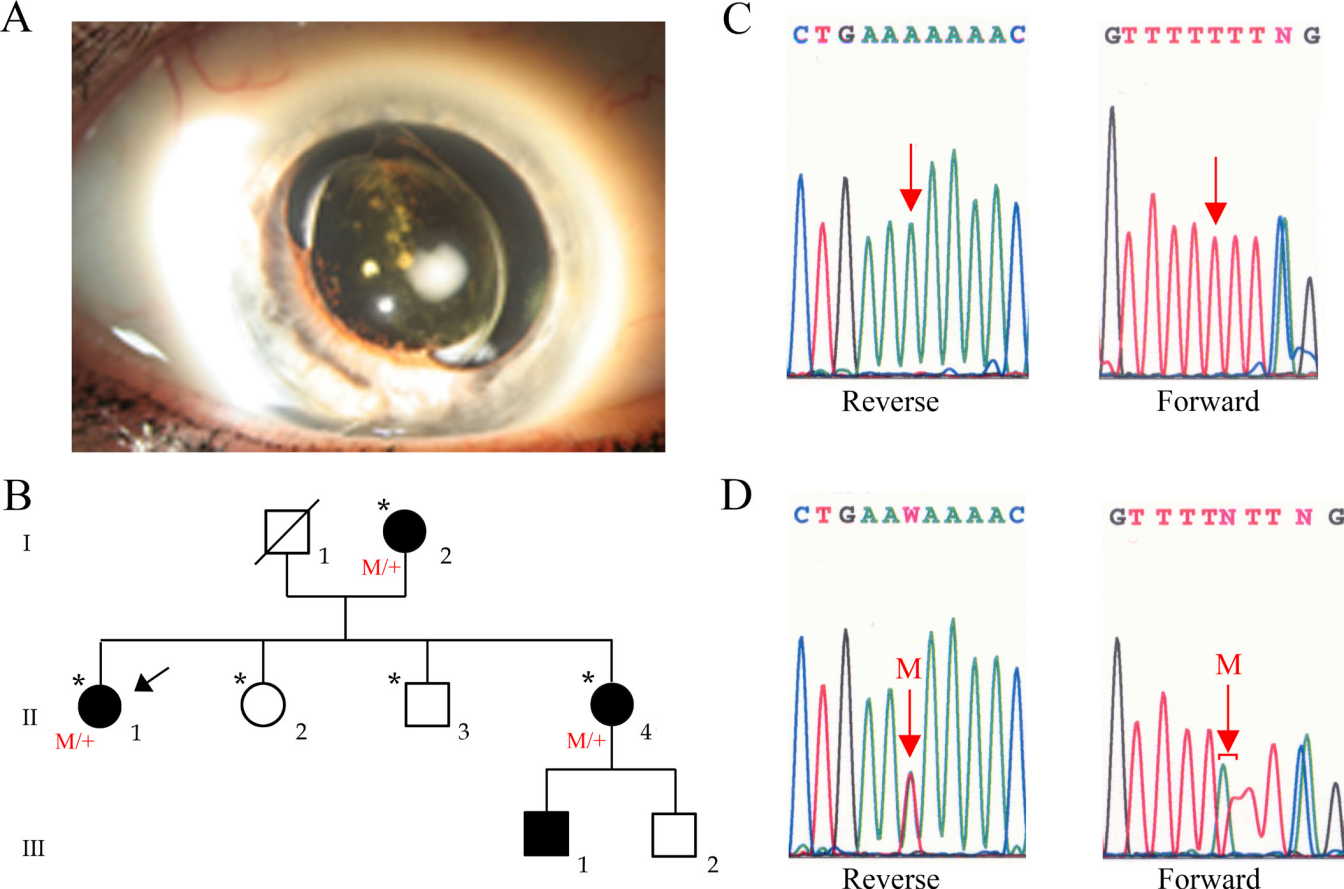
Ophthalmic and genetic analysis of a family with Wagner syndrome. **A**: Anterior segment photograph of the proband’s right eye (individual II:1) showing the synechiae between the intraocular lens and the iris, a temporally subluxated intraocular lens (IOL) as well as anterior and posterior capsular fibrosis. **B**: Pedigree of the family with Wagner syndrome. The arrow designates the proband, and the asterisks point to the family members examined and sequenced at our center. The symbol “M/+” designates patients with the mutation. **C**: Normal sequence chromatogram of the versican (*VCAN*) gene. **D**: Forward and reverse mutated sequence chromatograms (individual II:1) of the *VCAN* gene shows a heterozygous T to A substitution (red arrow with the letter “M”) at the sixth base of the splice acceptor site of intron 7 (c.4004–6T>A). Note that for all patients the mutated forward sequence was of poorer quality that of the reverse sequence.

The proband was a 45-year-old woman, born after a full-term pregnancy and normal delivery to non-consanguineous parents (the pedigree of the family is provided in Figure 1B). At the age of 30, the proband developed a bilateral nuclear cataract that required surgical removal at the age of 43. Several weeks after uncomplicated cataract surgery, she developed severe bilateral postoperative uveitis in both eyes responsible for bilateral anterior and posterior capsular fibrosis and intraocular lenses (IOLs) subluxation. Remission of the intraocular inflammation was obtained at first with topically administered steroid drops, but several attacks of uveitis progressively became refractory to treatment and were further responsible for macular cystoid edema in the left eye.

**Figure 2:**
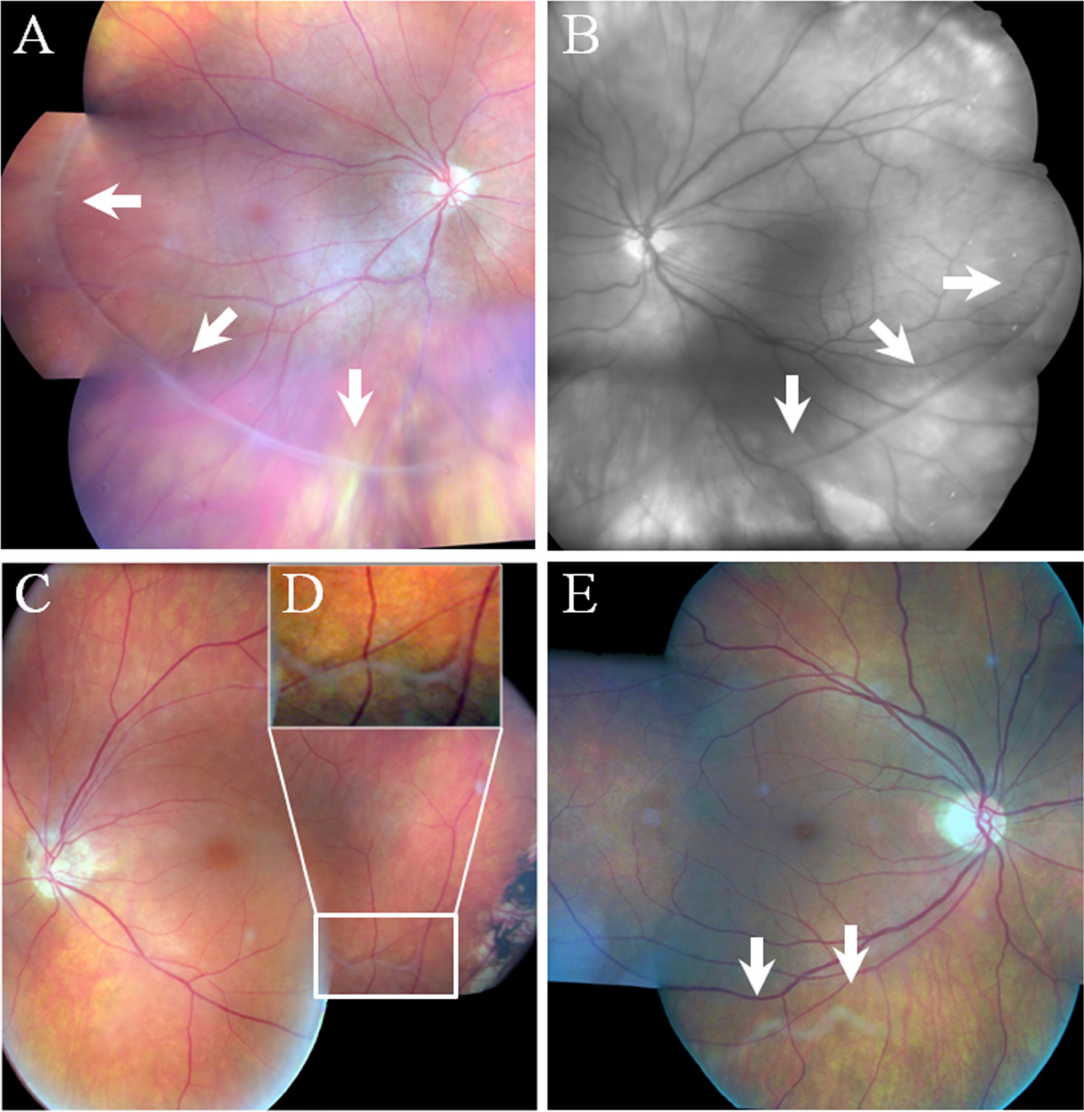
Composite fundus photograph showing several aspects of the avascular vitreous veil (arrows and white box), a hallmark feature of Wagner syndrome. **A**: The color fundus photograph of the proband’s right eye (individual II:1) shows a well-defined circumferential veil. **B**: Red-free fundus photograph of the proband’s left eye (Individual II:1) shows the vitreous veil as well as bright reflective areas corresponding to peripheral chorioretinal atrophy. **C**: Photograph of the proband’s mother left eye (individual I:2) also shows a barely detectable veil. **D**: Enlargement of the area within the box shows the veil firmly attached to the underlying retina. **E**: Photograph of the proband’s sister right eye (individual II:5) shows a barely detectable veil more easily seen in the inferotemporal retinal periphery.

When the patient was referred to our uveitis clinic, her visual acuity (VA) was 0.6 in the right eye and 0.1 in the left eye (Table 1). The intraocular pressure (IOP) was slightly elevated in both eyes (22 and 23 mmHg, respectively). The slit-lamp examination revealed the presence of inflammatory cells in both anterior chambers, though predominantly in the left eye. Laser flare meter values were 16±1 photons/ms in the right eye and 119.5±1.5 photons/ms in the left eye (normal values<10 photons/ms). Both eyes exhibited synechiae between the iris and the anterior capsulorhexis or the IOL as well as temporal subluxation of the lenses and prominent capsular fibrosis (Figure 1A). The dilated fundus examination revealed an optically empty vitreous, with peculiar vitreous cortex condensation found circumferentially at the equator level and detached from the underlying retina from the equator toward the retinal periphery (Figure 2A,B). Ectopic fovea responsible for clinically visible 20° exotropia (pseudostrabismus) as well as mild temporal dragging of the retinal vessels were noted. Finally, a degree of chorioretinal atrophy was found scattered at the posterior pole and at the retinal periphery. In addition, the left eye exhibited cystoid macular edema. Kinetic perimetry showed constricted peripheral isopters and a relative central scotoma. Automatic perimetry showed alterations consistent with glaucomatous defects. Full-field electroretinogram showed decreased a- and b-wave amplitudes, more pronounced for the photopic responses with no implicit time shift, consistent with cone-restricted dysfunction.

**Table 1:** Summary of clinical characteristics of the wagner family with the c.4004–6T>A *VCAN* mutation

Patient/Age	Visual acuity (Right/Left)	Refractive status	Uveitis	Cataract	Glaucoma	Optically empty vitreous with veils	Chorioretinal atrophy	Retinal detachment
I-2/72yrs	NLP/0.6	Pseudophakic	+	++	++	+	+	+
II-1/45 years	0.6/0.1	Pseudophakic	+++	+++	+	+++	++	-
II-4/48 years	0.8 /0.8	Mild myopia	-	++	-	+	+	-

NLP: no light perception; clinical severity is graded as mild (+), moderate (++) or severe (+++) according to the intensity and/or the early-onset nature of the manifestation

A comprehensive systemic clinical examination and an extensive evaluation for common causes of uveitis were negative. A periocular (subtenon) steroid injection to treat the cystoid macular edema in the left eye resulted in a good anatomic response, but VA did not improve. Controlling the intraocular pressure required two topically administered antiglaucoma medications, including a carbonic anhydrase inhibitor and a beta-blocker.

#### Individual I:2

The proband’s mother was 72 years old, with no previous systemic medical history or family history of ocular disease. She was diagnosed with bilateral exotropia early in childhood, which did not require any intervention (consistent with pseudostrabismus due to ectopic fovea). Retinal detachment occurred in the right eye at the age of 10, with subsequent total and irreversible loss of vision. She underwent a cataract extraction in the left eye at the age of 51. A preventive peripheral laser photocoagulation for peripheral retinal degeneration was performed before the cataract surgery procedure. Open angle glaucoma developed shortly after the procedure and required the administration of a single topical antiglaucoma medication (beta-blocker) to successfully control the intraocular pressure and prevent the further progression of the visual fields’ defects.

On examination, VA in the left eye was 0.6, and slit-lamp biomicroscopy revealed the presence of anterior chamber inflammatory cells. Laser cell flare meter values were 51±9.6 photons/ms. Mild posterior capsular opacification was noted, and IOP was within normal limits. The dilated fundus examination revealed an optically empty vitreous with a barely visible detached and condensed vitreous cortex at the level of the equator, more easily seen in the inferior and temporal retinal quadrant (Figure 2C,D).

#### Individual II:4

The proband’s sister was 48 years old with no history of ocular disease except mild myopia. On examination, her VA was 0.8 in both eyes, with an optical correction of −0.50 diopters. IOP and laser cell flare meter values were within normal limits. On slit-lamp examination, the anterior chamber was quiet, and the crystalline lens exhibited diffuse dot-like opacities as well as a nuclear cataract. The dilated fundus examination revealed an optically empty vitreous with a condensed vitreous cortex detached from the underlying retina at the level of the equator, more visible in the inferior and temporal quadrant (Figure 2E).

### Other individuals

The ophthalmologic examinations of unaffected individuals II:2 and II:3 were unremarkable. Individual III:3 declined to be examined at our center, but the patient’s medical records revealed a severe visual loss due to unilateral retinal detachment at the age of 20.

### Molecular analysis

We sequenced a set of six genes involved in various forms of exudative and degenerative familial vitreoretinopathies, including the 54 coding regions of *COL2A1*, the 15 coding regions of *VCAN*, the two coding regions of *FZD4*, the 23 coding regions of *LRP5*, the eight coding regions of *TSPAN12*, and the three coding regions of *NDP* from affected individual II:1 of this family. A total of 38 single nucleotide variations were detected; all are summarized in Table 2. After the variations referenced as single nucleotide polymorphisms (SNPs) located in intronic or in 5′- and 3′- untranslated regions in the dbSNP were excluded, only 18 changes remained (see Methods). Of these, 11 synonymous and six non-synonymous variants were identified in coding regions of *LRP5*, *VCAN*, and *COL2A1*, and one nucleotide substitution in the canonical splice acceptor site of *VCAN*. All synonymous and non-synonymous variants found in *LRP5*, *VCAN*, and *COL2A1* were already referenced in the dbSNP, and were found in our control population (heterozygous or homozygous state). These variants were therefore excluded as pathogenic variants. Finally, the only nucleotide variation corresponding to a new and unique change, not referenced in the dbSNP, not identified in control samples, and found to segregate with the disease status in this family was the heterozygous T to A substitution at the sixth base of the splice acceptor site of intron 7 of *VCAN*, c.4004–6T>A (Figure 1D).

**Table 2:** Summary of nucleotide variations identified

Gene symbol	cDNA position	Inheritance	Gene region	Exon	Translation impact	Ref dbSNP
FZD4	c.*4664T>C	Homo	3′ UTR	2		rs713065
FZD4	c.*2971T>C	Hetero	3′ UTR	2		rs10898563
FZD4	c.*1572G>C	Homo	3′ UTR	2		rs4944641
FZD4	c.*1298C>T	Homo	3′ UTR	2		rs3802892
LRP5	c.687–48A>T	Homo	intronic	4		rs10791978
LRP5	c.884–4T>C	Homo	intronic	5		rs314776
LRP5	c.1412+8G>A	Hetero	intronic	6		rs4988319
LRP5	c.1647T>C	Homo	exonic	8	p.F549F	rs545382
LRP5	c.2220C>T	Hetero	exonic	10	p.N740N	rs2306862
LRP5	c.2503+78A>G	Hetero	intronic	11		rs689179
LRP5	c.3237–52T>G	Hetero	intronic	15		rs554734
LRP5	c.3357G>A	Hetero	exonic	15	p.V1119V	rs556442
LRP5	c.3638–82C>T	Hetero	intronic	17		rs607887
LRP5	c.3989C>T	Hetero	exonic	18	p.A1330V	rs3736228
TSPAN12	c.*39C>T	Homo	3′ UTR	8		rs41622
TSPAN12	c.765G>T	Homo	exonic	8	p.P255P	rs41623
VCAN	c.348T>C	Hetero	exonic	3	p.T116T	rs12332199
VCAN	c.645A>G	Hetero	exonic	5	p.V215V	rs4470745
VCAN	c.4004–6T>A	Hetero	intronic	8		-
VCAN	c.4323G>A	Homo	exonic	8	p.Q1441Q	rs2548541
VCAN	c.4547A>G	Homo	exonic	8	p.K1516R	rs309559
VCAN	c.5477G>A	Homo	exonic	8	p.R1826H	rs188703
VCAN	c.5808T>C	Homo	exonic	8	p.G1936G	rs309557
VCAN	c.6723A>G	Homo	exonic	8	p.R2241R	rs160279
VCAN	c.6902T>A	Homo	exonic	8	p.F2301Y	rs160278
VCAN	c.8809G>T	Homo	exonic	8	p.D2937Y	rs160277
VCAN	c.9075G>A	Hetero	exonic	8	p.T3025T	rs113014073
VCAN	c.9266–97C>A	Hetero	intronic	9		rs148382459
VCAN	c.9494–63T>A	Hetero	intronic	11		rs6873404
VCAN	c.9882C>T	Homo	exonic	14	p.V3294V	rs308365
COL2A1	c.2301+72C>T	Hetero	intronic	34		rs12811832
COL2A1	c.2050–49G>T	Hetero	intronic	32		rs11168338
COL2A1	c.1527+88T>C	Hetero	intronic	23		rs1635544
COL2A1	c.1023+108T>C	Hetero	intronic	16		rs1635534
COL2A1	c.1023+84C>A	Hetero	intronic	16		rs1793915
COL2A1	c.654+15T>G	Hetero	intronic	9		rs1034762
COL2A1	c.85+18C>G	Hetero	intronic	1		rs3803184
COL2A1	c.25A>T	Hetero	exonic	1	p.T9S	rs3803183

### In silico predictions of c.4004–6T>A mutation on versican messenger ribonucleic acid processing

We performed in silico analyses with the software previously used by Kloeckener-Gruissem et al. to evaluate the putative effects of our novel c.4004–6T>A mutation on splicing compared with all WS splicing mutations published to date [12]. These bioinformatics data were compared with the corresponding experimental *VCAN* messenger RNA (mRNA) analyses previously reported (Table 3 and references herein). All seven variants located within the canonical splice site dinucleotides that flank exon 8 of the *VCAN* gene were predicted to be spliceogenic, which was in line with in vitro *VCAN* mRNA analyses [3-5,7,8,10-13]. The potential effects of the two variants at −5 (c.4004–5T>A and T>C) on splicing were more challenging, as they did not drastically decrease the strength score of intron 7’s acceptor splice site, as predicted by MaxtEnt Scan and NetGene2. Moreover, those effects were considered not damaging by three other computational splice site algorithms. Nevertheless, these two variants at −5 were experimentally proven spliceogenic in peripheral blood leukocytes from mutation carriers, demonstrating that bioinformatics prediction tools are not yet fully accurate and can underestimate the importance of a sequence variation in the splicing process. In silico data for our novel variant at −6 (c.4004–6T>A) showed splice site predictions similar to those found for mutations at −5, suggesting the high likelihood that our variant at −6 might impair the proper processing of *VCAN* mRNA. Of note, the splice site strength score of intron 7 obtained for our variant at −6 was significantly lower than those found for variants at −5 with the NetGene2 algorithm.

**Table 3:** In silico analyses of published VCAN splice-site mutations.

Sequence and Reference	Position	Function	Experimental mRNA confirmation	Human Splicing Finder***^1^***	MaxEnt Scan***^2^***	NNSplice***^3^***	NetGene2***^4^***	ESEfinder***^5^***
Reference NG_012682	intron 7/exon8	Acceptor		94	9.88	0.99	0.97	12.23
c.4004 −1 G>C [12]	intron 7/exon8	Acceptor	Yes	65.05	1.82	-	-	-
c.4004 −1 G>A [10,11]	intron 7/exon8	Acceptor	Yes	65.05	1.13	-	-	-
c.4004 −2 A>G [3]	intron 7/exon8	Acceptor	Yes	65.05	1.93	-	-	-
c.4004 −2 A>T [4]	intron 7/exon8	Acceptor	Yes	65.05	1.52	-	-	-
c.4004 −5 T>C [10]	intron 7/exon8	Acceptor	Yes	93.11	8.14	0.99	0.94	11.8
c.4004 −5 T>A [10]	intron 7/exon8	Acceptor	Yes	90.44	7.7	0.97	0.83	9.69
c.4004 −6 T>A This study	intron 7/exon8	Acceptor	No	91.23	7.83	0.95	0.43	9.77
Reference NG_012682	exon8/intron8	Donor		97.66	10.77	1	1	10.47
c.9265 +1 G>T [8]	exon8/intron8	Donor	No	70.83	2.26	-	-	-
c.9265 +1 G>A [5,7,13]	exon8/intron8	Donor	Yes	70.83	2.58	-	-	-
c.9265 +2 T>A [12]	exon8/intron8	Donor	Yes	70.83	2.58	-	-	-

## Discussion

We report a new *VCAN* splicing mutation causing typical Wagner syndrome vitreous features associated with unexplained intraocular inflammatory manifestations. This nucleotide substitution, c.4004–6T>A, occurred at the sixth position of the acceptor splice site of intron 7 where splice disease-mutations at the fifth, the second, and the first base have been previously reported [3,4,10-12]. Although a *VCAN* transcript analysis could not be performed in this kindred, the concordance of this new *VCAN* gene mutation with clinically affected members, coupled with the fact that this new mutation occurred at a splice consensus site where numerous causative WS mutations have been previously reported, establishes that this new mutation is most likely deleterious. Furthermore, the absence of pathogenic variants in other genes known to be involved in hereditary vitreoretinopathies provides further evidence of the causative role of this unique *VCAN* mutation, not identified in any SNP database.

Although it has been now clearly demonstrated that WS disease splicing mutations cause aberrant increased expression of V2/V3 *VCAN* isoforms in peripheral blood leukocytes or in skin fibroblasts, why the clinical phenotype is restricted to ocular manifestations remains elusive. However, splicing alterations observed in non-affected tissues, such as blood or skin, might not fully reflect those in tissues at risk in Wagner syndrome. The first WS causative variants identified were intronic variants located in the canonical splice site dinucleotides. These variants are commonly assumed to be highly pathogenic based on their position alone. However, intronic sequence variations, whether they occur in close proximity to these sites or more deeply in intronic regions, can disrupt other splice motifs or auxiliary cis-regulatory elements responsible for splicing defects. These defects could be pathogenic only in specific tissues under the influence of a set of regulatory trans-acting splicing factors. Therefore, the identification of this novel *VCAN* splice mutation at position −6 supports the idea that the mutations occurring at deeper intronic positions than the canonical dinucleotides of introns 7 and 8 of *VCAN* may be of clinical relevance, as exemplified for the Chinese family with Wagner syndrome reported by Mukhopdhyay et al., for which the causal variant has not yet been identified [10].

The first report of Wagner syndrome–related uveitis consisted of a father and his daughter who had several spontaneous uveitis attacks that flared up after the phacoemulsification procedure, with early-onset prominent posterior capsular opacification [7]. In 2011, we reported the second family with WS revealed by longstanding spontaneous uveitis in the proband and uncontrolled postoperative inflammation in other affected members of this family [4]. In the third family with WS with uveitis reported here, none of the affected family members of this kindred had spontaneous uveitis. The proband presented bilateral chronic and severe uveitis following a surgery procedure for early-onset cataract, and her mother showed chronic low-grade uveitis, probably also triggered by cataract surgery performed several years before.

Several lines of evidence from the basic research on different physiologic functions of versican have recently highlighted the pivotal role of versican in inflammation [24]. Indeed, versican is a complex extracellular matrix protein with pleiotropic effects due to its ability to bind multiple partners, including proinflammatory cytokines [25,26]. Although it has been shown that splicing mutations cause tissue-specific versican isoform imbalance, the mechanisms explaining either uveitis or the exclusively ocular phenotype are poorly understood [10,12].

Altogether, the clinical inflammatory manifestations in patients with WS and the inflammatory properties of versican contribute to suggest that intraocular inflammation could belong to the clinical spectrum of Wagner syndrome. However, the limited number of families with WS reported to date does not allow us to draw definitive conclusions regarding the possible association between WS and uveitis. The characterization of more families is needed, especially from uveitis clinics, to better clarify the frequency of inflammatory complications associated with WS. Although uveitis varies in severity in patients with WS, it could nonetheless be responsible for significant visual morbidity in addition to chorioretinal atrophy or retinal detachment. Therefore, we suggest that clinicians consider WS as a possible diagnosis in patients with unexplained uveitis.

## Appendix 1. Primer sequences for pcr amplification of COL2A1 gene.

To access the data, click or select the words “Appendix 1.”

## References

[1] Wagner H (1938). Ein bisher unbekanntes Erbleiden des Auges (degeneratio hyaloideo-retinalis hereditaria), beobachtet im Kanton Zurich.. Klin Monatsbl Augenheilkd.

[2] Graemiger RA, Niemeyer G, Schneeberger SA, Messmer EP (1995). Wagner vitreoretinal degeneration. Follow-up of the original pedigree.. Ophthalmology.

[3] Miyamoto T, Inoue H, Sakamoto Y, Kudo E, Naito T, Mikawa T, Mikawa Y, Isashiki Y, Osabe D, Shinohara S, Shiota H, Itakura M (2005). Identification of a novel splice site mutation of the CSPG2 gene in a Japanese family with Wagner syndrome.. Invest Ophthalmol Vis Sci.

[4] Brézin AP, Nedelec B, Barjol A, Rothschild PR, Delpech M, Valleix S (2011). A new VCAN/versican splice acceptor site mutation in a French Wagner family associated with vascular and inflammatory ocular features.. Mol Vis.

[5] Rothschild PR, Audo I, Nedelec B, Ghiotti T, Brézin AP, Monin C, Valleix S (2013). De Novo Splice Mutation in the Versican Gene in a Family With Wagner Syndrome.. JAMA Ophthalmol.

[6] Zech JC, Morle L, Vincent P, Alloisio N, Bozon M, Gonnet C, Milazzo S, Grange JD, Trepsat C, Godet J, Plauchu H (1999). Wagner vitreoretinal degeneration with genetic linkage refinement on chromosome 5q13-q14.. Graefes Arch Clin Exp Ophthalmol.

[7] Meredith SP, Richards AJ, Flanagan DW, Scott JD, Poulson AV, Snead MP (2007). Clinical characterisation and molecular analysis of Wagner syndrome.. Br J Ophthalmol.

[8] Ronan SM, Tran-Viet KN, Burner EL, Metlapally R, Toth CA, Young TL (2009). Mutational hot spot potential of a novel base pair mutation of the CSPG2 gene in a family with Wagner syndrome.. Arch Ophthalmol.

[9] Fryer AE, Upadhyaya M, Littler M, Bacon P, Watkins D, Tsipouras P, Harper PS (1990). Exclusion of COL2A1 as a candidate gene in a family with Wagner-Stickler syndrome.. J Med Genet.

[10] Mukhopadhyay A, Nikopoulos K, Maugeri A, de Brouwer AP, van Nouhuys CE, Boon CJ, Perveen R, Zegers HA, Wittebol-Post D, van den Biesen PR, van der Velde-Visser SD, Brunner HG, Black GC, Hoyng CB, Cremers FP (2006). Erosive vitreoretinopathy and wagner disease are caused by intronic mutations in CSPG2/Versican that result in an imbalance of splice variants.. Invest Ophthalmol Vis Sci.

[11] Chen X, Zhao K, Sheng X, Li Y, Gao X, Zhang X, Kang X, Pan X, Liu Y, Jiang C, Shi H, Rong W, Chen LJ, Lai TY, Wang X, Yuan S, Liu Q, Vollrath D, Pang CP, Zhao C (2013). Targeted sequencing of 179 genes associated with hereditary retinal dystrophies and 10 candidate genes identifies novel and known mutations in patients with various retinal diseases.. Invest Ophthalmol Vis Sci.

[12] Kloeckener-Gruissem B, Neidhardt J, Magyar I, Plauchu H, Zech JC, Morlé L, Palmer-Smith SM, Macdonald MJ, Nas V, Fry AE, Berger W (2013). Novel VCAN mutations and evidence for unbalanced alternative splicing in the pathogenesis of Wagner syndrome.. Eur J Hum Genet.

[13] Kloeckener-Gruissem B, Bartholdi D, Abdou MT, Zimmermann DR, Berger W (2006). Identification of the genetic defect in the original Wagner syndrome family.. Mol Vis.

[14] Poulter JA, Ali M, Gilmour DF, Rice A, Kondo H, Hayashi K, Mackey DA, Kearns LS, Ruddle JB, Craig JE, Pierce EA, Downey LM, Mohamed MD, Markham AF, Inglehearn CF, Toomes C (2010). Mutations in TSPAN12 cause autosomal-dominant familial exudative vitreoretinopathy.. Am J Hum Genet.

[15] Qin M, Hayashi H, Oshima K, Tahira T, Hayashi K, Kondo H (2005). Complexity of the genotype-phenotype correlation in familial exudative vitreoretinopathy with mutations in the LRP5 and/or FZD4 genes.. Hum Mutat.

[16] Desmet FO, Hamroun D, Lalande M, Collod-Beroud G, Claustres M, Beroud C (2009). Human Splicing Finder: an online bioinformatics tool to predict splicing signals.. Nucleic Acids Res.

[17] Yeo G, Burge CB (2004). Maximum entropy modeling of short sequence motifs with applications to RNA splicing signals.. J Comput Biol.

[18] Eng L, Coutinho G, Nahas S, Yeo G, Tanouye R, Babaei M, Dork T, Burge C, Gatti RA (2004). Nonclassical splicing mutations in the coding and noncoding regions of the ATM Gene: maximum entropy estimates of splice junction strengths.. Hum Mutat.

[19] Reese MG, Eeckman FH, Kulp D, Haussler D (1997). Improved splice site detection in Genie.. J Comput Biol.

[20] Hebsgaard SM, Korning PG, Tolstrup N, Engelbrecht J, Rouze P, Brunak S (1996). Splice site prediction in Arabidopsis thaliana pre-mRNA by combining local and global sequence information.. Nucleic Acids Res.

[21] Brunak S, Engelbrecht J, Knudsen S (1991). Prediction of human mRNA donor and acceptor sites from the DNA sequence.. J Mol Biol.

[22] Smith PJ, Zhang C, Wang J, Chew SL, Zhang MQ, Krainer AR (2006). An increased specificity score matrix for the prediction of SF2/ASF-specific exonic splicing enhancers.. Hum Mol Genet.

[23] Cartegni L, Wang J, Zhu Z, Zhang MQ, Krainer AR (2003). ESEfinder: A web resource to identify exonic splicing enhancers.. Nucleic Acids Res.

[24] Zhang Z, Miao L, Wang L (2012). Inflammation amplification by versican: the first mediator.. Int J Mol Sci.

[25] Theocharis AD (2008). Versican in health and disease.. Connect Tissue Res.

[26] Handley CJ, Samiric T, Ilic M (2006). Structure, metabolism, and tissue roles of chondroitin sulfate proteoglycans.. Adv Pharmacol.

